# Interleukin-6 and Vitamin D Status during High-Intensity Resistance Training in Patients with Chronic Kidney Disease

**DOI:** 10.1155/2014/176190

**Published:** 2014-04-02

**Authors:** Stig Molsted, Pia Eiken, Jesper L. Andersen, Inge Eidemak, Adrian P. Harrison

**Affiliations:** ^1^Department of Cardiology, Nephrology & Endocrinology, Nordsjællands University Hospital, Dyrehavevej 29, 3400 Hillerød, Denmark; ^2^Faculty of Health and Medical Sciences, Copenhagen University, Blegdamsvej 3, 2200 Copenhagen N, Denmark; ^3^Institute of Sports Medicine Copenhagen, Bispebjerg Hospital, University of Copenhagen, Bispebjerg Bakke 23, 2400 Copenhagen NV, Denmark; ^4^Department of Nephrology P, Rigshospitalet, Copenhagen University Hospital, Blegdamsvej 9, 2100 Copenhagen Ø, Denmark; ^5^Department of Basic Animal and Veterinary Sciences, Faculty of Health and Medical Sciences, Copenhagen University, Grønnegårdsvej 15, 1870 Frederiksberg C, Denmark

## Abstract

*Background*. The aim of this study was to investigate IL-6 and 25-hydroxyvitamin D (25-OH D) associations with muscle size and muscle function in dialysis patients. *Methods*. Patients were included in a 16-week control period followed by 16 weeks of high-intensity resistance training thrice weekly. IL-6 and 25-OH D were analysed after an over-night fast. Muscle fibre size was analysed in biopsies from * m. vastus lateralis*. Muscle power was tested using a Leg Extensor Power Rig. * Results*. Patients (*n* = 36) with IL-6 ≥ 6.49 pg/ml (median) were older and had decreased muscle power and a reduced protein intake (*P* < 0.05) compared with patients with IL-6 < 6.49 pg/ml. IL-6 was not associated with muscle fibre size. Vitamin D deficiency (25-OH D < 50 nmol/l) was present in 51% of the patients and not associated with muscle power. IL-6 remained unchanged during the training period, whilst muscle power increased by 20–23% (*P* < 0.001). * Conclusion*. Elevated IL-6 values were associated with decreased muscle power but not with decreased muscle fibre size. Half of the patients were suffering from vitamin D deficiency, which was not associated with muscle power. IL-6 was unchanged by high-intensity resistance training in dialysis patients in this study.

## 1. Introduction

Chronic kidney disease (CKD) can arise as the result of several diseases, among them diabetes. When renal function is reduced to approximately 10% of the healthy renal capacity of an individual, dialysis therapy or kidney transplantation is needed in order to keep the patient alive. The majority of patients with CKD are treated with haemodialysis thrice weekly or daily by means of peritoneal dialysis.

Low-grade systemic inflammation with increased levels of proinflammatory cytokines is common in patients with CKD [1]. The origin of this low-grade inflammation in patients with CKD is not clear, but it could be a complication related to either impaired kidney function or dialysis treatment* per se,* or both [2]. The low-grade inflammation is likely to contribute to the high mortality rate in dialysis patients via an elevated protein energy waste [3, 4]. Whilst protein energy waste increases the mortality risk, it may also affect muscle mass negatively and thereby reduce the patients' muscle strength. Thus, a direct link between low-grade inflammation and a decrease in muscle strength impairing physical performance and reduced ability to perform daily activities is likely.

In subjects not suffering from CKD, low-grade inflammation is associated with physical inactivity [5]. As physical inactivity is a common problem in dialysis patients, the question remains as to whether low-grade inflammation in these patients can be reduced by physical exercise. Previous studies have not shown any effect of exercise training on low-grade inflammation in dialysis patients, but the results may be affected by the training modalities and programs used.

In parallel with low-grade inflammation, vitamin D deficiency determined as low values of 25-hydroxyvitamin D (25-OH D) is common in patients with CKD [6–8]. The vitamin D deficiency in patients with CKD arises from a diminished exposure to sunlight and through malnutrition. Vitamin D deficiency has serious implications in terms of reduced muscle function [9] and an increased mortality risk in dialysis patients [6]. Furthermore, it is likely that an impact of vitamin D deficiency on muscle function may be present in CKD patients even before dialysis therapy is needed [10]. The causality between vitamin D deficiency and reduced muscle size is not clear, but vitamin D deficiency may impair protein synthesis leading to muscle waste [11].

Thus, low-grade inflammation and vitamin D deficiency may have a negative impact on muscle mass and muscle function in dialysis patients. The aim of this study was to investigate the effect of resistance training on the proinflammatory factor IL-6 as well as the impact of 25-OH D on muscle size in dialysis patients.

## 2. Material and Methods

The data from the present study were obtained as part of a recent study of effects of resistance training in dialysis patients [12]. The patients were recruited from three dialysis centres in the Capital Region, Demark, to a control period of 16 weeks without any intervention followed by an intervention period of 16 weeks with resistance training. The study was performed with three cohorts over three periods of time. To be included, the participants had to be above 18 years of age and undergoing haemodialysis or peritoneal dialysis for more than three months. Exclusion criteria were insulin treatment, severe diabetic retinopathy, leg amputation, severe peripheral polyneuropathy, dementia, and an inability to speak Danish. The capacity of the study program allowed all included patients to participate.

Comorbidity level was assessed using the Index of Coexistent Disease [13]. Food intake was assessed using a modified 7-day version of the Inter99 food frequency questionnaire [14] and energy intake was determined with the aid of Master Diætist Data software (Anova A/S, Holte, Denmark).

The tests were conducted with the same relation to the dialysis schedule at the three tests. Informed consent was obtained from all patients and the local ethical committee approved the protocol (H-D-2008-124). The study was registered on controlled-trials.com no. ISRCTN72099857.

### 2.1. Training Program

The training consisted of supervised progressive high-intensity resistance training three times a week for 16 weeks [12]. The training began with 5 minutes of warm-up followed by up to 5 sets of dynamic leg press, dynamic leg extension, and dynamic leg curl, respectively. The rest period between each set was 60–90 seconds (the time between repetitions was not regulated). During the intervention period the load was increased and the number of repetitions decreased correspondingly from 15 repetitions in the beginning of the intervention period to 6 repetitions in the final period (see Table 1). Every set was performed to exhaustion. The progression during the training period was adjusted according to changes in 1 repetition maximum (RM). The 1RM was tested six times in the exercise machines used in the training program (Technogym, Rotterdam, The Netherlands) prior to the subperiods presented in Table 1 (first test day 1, week 1). After 5 minutes of warm-up, the participants completed a full concentric knee extension from a 90-degree flexionwhile sitting in an upraised position to test knee extension 1RM. In the 1RM test, the first attempt was conducted close to an estimated maximum strength and the following attempts were conducted with an increased resistance of 2.5 to 10 kg. The muscle strength test was conducted with a maximum of 6 attempts and the highest value was accepted as the result. Rest period between each attempt was 60 seconds. Every participant conducted the test with the same machine position at “pretraining” and at “posttraining” test. The load in the leg press exercise was also determined using the previously described testing protocol. In the leg press test the participants completed a full concentric knee extension from 90-degree flexion in the knee and hip joints. The knee extension and the leg press tests were conducted on the same days in the presented order. The training intensity was calculated using the relations 6RM ~90% of 1RM; 8RM ~86% of 1RM; 10RM ~82% of 1RM; 12RM ~77% of 1RM; and 15RM ~71% of 1RM. Three patients did not feel comfortable during the high loaded 1RM tests, which were replaced by 6RM tests used in the intensity progression. The 1RM tests were conducted on training days before the exercises. The intensity progression during the 16 weeks of training was in addition to the 1RM tests based on increased muscle strength observed between the 1RM tests during the intervention period. Thus, as the patients were able to exceed the estimated number of repetitions according to the training protocol, the load was elevated immediately. In the leg curl exercise, the intensity was not based on 1RM tests but on the number of repetitions performed to exhaustion during the training program.

Whilst the predefined number of repetitions during every training session was completed in the first two to three sets, the number of repetitions usually declined in the following sets as exhaustion was achieved earlier.

### 2.2. Muscle Power and Physical Function

Leg extensor power was measured using the Nottingham Leg Extensor Power Rig. The patients sat in an upright position with their arms folded across their chest and with their active leg towards the push-pedal in front of the seat, which made the direction of movement almost horizontal. The subject was instructed to push the pedal as hard and as fast as possible. The measurement was repeated at least five times and until no further improvement could be recorded on two consecutive occasions. The data were recorded, computed, and expressed in watts (W) using the Leg Rig software package (PC214E; University of Nottingham, Medical Faculty Workshops, Queen's Medical Centre, Nottingham, UK).

Physical function was measured using the Chair Stand Test from the Senior Fitness Test [15]. The test required the patients to rise to a full standing position and return to a seated position as frequently as possible within a 30-second time frame whilst maintaining their arms folded across their chest at all times.

### 2.3. Muscle Fibre Analyses

Muscle biopsies were obtained from the midregion of* m. vastus lateralis* on nonhaemodialysis days through a five-millimetre incision using a Bergström biopsy needle. Serial sections (10 *μ*m) of the muscle biopsy samples were cut and myofibrillar ATPase histochemistry was performed at pH 9.40 after preincubation at pH values 4.37, 4.60, and 10.30 [16]. Computer image analysis was performed using an image analysis system. Fibres were subsequently classified as type 1, type 2A, and type 2X [17]. Fibre type sizes were only performed on the two major fibre types (1 and 2). Only truly horizontally cut fibres were analyzed.

### 2.4. Blood Tests

Interleukin-6, 1,25-dihydroxyvitamin D (1,25-OH_2_ D), and 25-OH D were analysed from blood taken after an overnight fast and a minimum of 18 hours after the last training session.

Plasma IL-6 was analysed using an ECLIA kit (Roche Diagnostics GmbH, Germany). According to the manufacturer, the limit of detection for this kit was 1.5 pg/mL, and the kit has been found to show no cross-reaction with such substances as IL-1*α*, IL-1*β*, IL-2, IL-3, IL-4, IL-8, IFN-*γ*, and TNF-*α*. The analyses were performed at the Department of Clinical Biochemistry, Aarhus University Hospital, Denmark.

Serum 25-OH D was analysed using an immunological method that measures both vitamin D 3 and D 2 [18]. Analyses were performed at the Department of Clinical Biochemistry, Aarhus University Hospital, Denmark. The lower limit of detection for this analysis was 10 nmol/L. D vitamin status was categorized as being either normal (25-OH D ≥ 50 nmol/L) or deficient (25-OH D < 50 nmol/L) according to the KDIGO guidelines of 2012 [19]. Albumin, haemoglobin, C-reactive protein (CRP), and bicarbonate tests were collected from the patients' clinical practice and were analysed in the laboratories of the hospitals comprising those servicing the Capital Region.

### 2.5. Statistical Analyses

Data distributions were tested using a Shapiro-Wilk Test. In analyses of variables with nonnormal data distributions nonparametric statistics were used. Binary correlations were tested using the Spearman Test. The Wilcoxon Signed Ranks Test or paired Student's* t*-test was used to test for differences between the baseline test and pretraining and between pre- and posttraining and also to compare the differences during the control period with any difference during the training period. The Mann-Whitney Test or unpaired Student's *t*-test was used to compare groups of patients. Age adjustments were performed using linear and logistic regression, and entered analyses and variables were log-transformed if the residuals were not normally distributed. Data are presented as the mean ± standard error of the mean (SEM), count, or percentages. All tests were two-tailed at a significance level of *P* ≤ 0.05.

## 3. Results

Thirty-six patients were included and 23 patients completed the intervention. Six patients dropped out during the control period and three patients dropped out during the training period due to problems not related to training. Four patients completed the intervention but were not retested due to illness, change of dialysis modality, or problems arising during blood sampling. Two patients were not included in the IL-6 measurements and one patient was not included in the 25-OH D measurements owing to insufficient plasma collection.

Clinical characteristics are presented in Table 2. There was no gender difference for IL-6 (females 8.0 ± 2.5 versus males 9.0 ± 1.4 pg/mL, *P* = 0.290). Interleukin-6 was positively correlated with age (*r* = 0.458, *P* = 0.006) and CRP (*r* = 0.693, *P* = 0.001). At baseline, 51% of the patients were vitamin D deficient. There was no gender difference for 25-OH D (female 62.0 ± 10.1 versus male 59.1 ± 7.6 nmol/L, *P* = 0.987).

Clinical data in patients with reduced or elevated IL-6 values (split on the median 6.49 pg/mL) are presented in Table 3. Patients with elevated IL-6 values (≥6.49 pg/mL) were significantly older, ingested fewer grams of protein·day^−1^·kilo^−1^ body weight, and had impaired muscle power compared to those with reduced IL-6 values. When muscle power was age and gender adjusted, low muscle power in the left leg remained associated with elevated IL-6 values (*P* = 0.042), whereas impaired muscle power in the right leg tended to be associated with elevated IL-6 values (*P* = 0.082).

Mean IL-6 remained stable throughout the control and training period (Table 5). When the patients were split on the IL-6 median prior to training (7.4 pg/mL), reduced baseline IL-6 values (4.8 ± 0.5 pg/mL) and elevated baseline IL-6 values (21.9 ± 4.8 pg/mL) were unchanged after the training period (data not shown).

The IL-6 values tested prior to and during the training period did not correlate with changes in muscle power or muscle morphology during the training period (data not shown).

Plasma albumin was elevated in patients with normal 25-OH D compared to those with vitamin D deficiency (Table 4). Patients with normal 25-OH D had a reduced percentage of type 2X muscle fibres and an increased type 1 muscle fibre size. When 25-OH D was age and season adjusted, normal 25-OH D correlated with the reduced percentage of type 2X muscle fibres (*P* = 0.012) and a reduction in type 1 muscle fibre size (*P* = 0.013).

**Figure 1:**
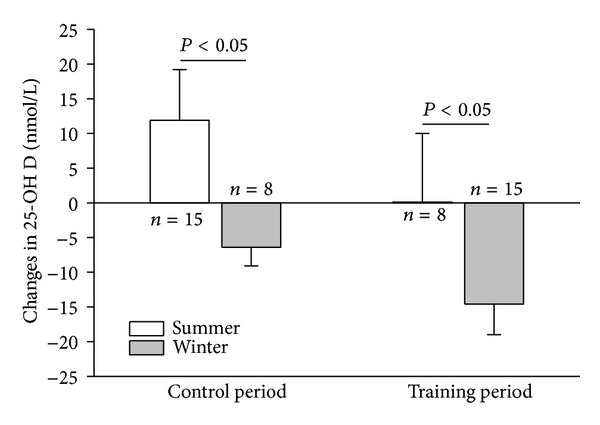
Changes in 25-OH D during the control period and the training period.

25-OH D was decreased after the training period (Table 5). In Figure 1, 25-OH D changes in the control and training period were stratified according to summer (April to September) and winter (October to March) periods. During the control and training periods, 25-OH D changed according to the season: during the summer, 25-OH D increased, and during the winter, the 25-OH D values decreased.

The 25-OH D status before and during the training period was not associated with changes in muscle power or muscle morphology during the training period (data not shown). However, decreased 25-OH D during the training period was correlated with an increase in the size of type 2 muscle fibres (*r* = −0.582, *P* = 0.009).

As previously shown, muscle power remained unchanged during the control period and increased during the training period (left leg from 2.01 ± 0.21 to 2.48 ± 0.20 W/kg, *P* < 0.001, and right leg from 2.11 ± 0.20 to 2.64 ± 0.19 W/kg, *P* < 0.001) [12]. Physical function determined by the Chair Stand Test remained also unchanged during the control period (from 15.2 ± 1.0 to 15.4 ± 1.3 repetitions, *P* = 0.164) and increased during the training period (to 18.7 ± 1.7 repetitions, *P* < 0.001) [12]. Muscle power changes during the training period were not correlated with IL-6 or 25-OH D (data not shown). As shown previously [20], mean muscle fibre size was not changed significantly between the pretraining and the posttraining tests (type 1: 4216 ± 311 versus 4427 ± 392 *μ*m^2^ and type 2: 3185 ± 233 *μ*m^2^ versus 3606 ± 275 *μ*m^2^, resp.). Body weight increased during the training period from 72.8 ± 3.7 to 73.9 ± 3.8 kg, *P* = 0.05.

## 4. Discussion

The main finding in this study was that 16 weeks of resistance training did not affect the level of the circulating proinflammatory cytokine IL-6 in a well-nourished sample of dialysis patients with relatively high values of plasma albumin. In cross-sectional analyses an elevated level of IL-6 was associated with impaired muscle power and a reduced protein intake. In addition, half of the patients were found to be suffering from vitamin D deficiency.

The IL-6 mean value was comparable with values reported in other studies focusing on skeletal muscle and exercise training in dialysis patients [21, 22]. In this study, patients with the most pronounced elevation of IL-6 had reduced muscle power, whereas no difference was found in muscle fibre size in-between patients with elevated or reduced IL-6 levels. Furthermore, an elevated IL-6 level was not found to affect the lack of muscle hypertrophy after training in this study. However, a previous study has suggested that low-grade inflammation disturbs the balance between protein breakdown and synthesis and is thereby associated with muscle size in dialysis patients [21]. The lack of muscle hypertrophy after the present resistance-training program may be the result of several disturbing conditions including low-grade inflammation. Uraemia [23], dialysis* per se* [24], insulin resistance [25], anabolic hormone deficiency [26], and acidosis [27] are factors that contribute to a negative protein balance making the catabolic mechanism multifactorial. Thus, even though an elevated IL-6 level was not found to be a limiting factor in this study, it may still play a role in combination with other factors in the catabolism of muscle in dialysis patients.

The literature reports that exercise training may decrease elevated levels of circulating IL-6 in subjects without CKD [5, 28, 29] and in patients with CKD prior to dialysis [30]. Thus, we hypothesized that resistance training would decrease elevated IL-6 in dialysis patients. Whilst the mean value of IL-6 in all patients remained unchanged after training, this was also true for the subgroup of patients with the highest IL-6 levels. The fact that IL-6 levels were unchanged after the resistance training in our patients is supported by two other trials on dialysis patients [22, 31]. However, a potential response of exercise training in terms of a decrease in IL-6 may depend on training modality, dose, and intensity [28]. Whilst resistance training was used in the present and the two other previously mentioned studies [22, 31], a pilot study tested the effect of aerobic exercise on inflammation in patients treated with dialysis and also found IL-6 to be unchanged [32]. Based on the present and previous results [22, 31, 32], we find no reason to believe that exercise training is effective in combating elevated IL-6 values in dialysis patients. However, we cannot exclude that a higher dose of training could be effective and future studies may change the present conclusions. On the other hand, the lack of effect of exercise on IL-6 may be explained by the origin of the low-grade inflammation. As CKD and the dialysis treatment are important contributors to low-grade inflammation [33], effects of exercise training on IL-6 may not be possible to achieve in this patient group due to the nature of the chronic disease and the indispensable dialysis treatment.

It is well documented that exercise induces acute increased levels of circulating IL-6, which is stimulated by contracting muscle [5, 28]. Interleukin-6 therefore not only is a cytokine but also is recognized as a myokine with an anti-inflammatory capacity [5]. In the present study it is unknown as to whether the posttraining levels of IL-6 were affected by any acute rise after the training sessions. However, as the lowest IL-6 values also remained unchanged after the training period, an acute effect of exercise training on IL-6 seems likely not to have affected the results.

Vitamin D values were in line with data reported in other studies on dialysis patients [6, 7, 9, 34]. Half of the patients were suffering from vitamin D deficiency. The 25-OH D values changed according to the season and may likely be the result of an increase in exposure to sunlight. The seasonal changes of 25-OH D were anticipated based on a previous finding [34].

Vitamin D deficiency in CKD patients is treated to prevent secondary hyperparathyroidism and osteomalacia. In addition, treatment with 1,25-dihydroxyvitamin D has in a retrospective cross-sectional study been suggested to be associated with greater muscle size and muscle strength in dialysis patients [10]. However, as the authors did not report any vitamin D data and only presented the patients as being treated or not with vitamin D, it is unknown if the vitamin D values were correlated with muscle size and strength. One such connection was not supported by the results from our study. Furthermore, it has also been suggested that vitamin D status in dialysis patients is associated with the level of physical activity [34]. In this study 25-OH D decreased during the resistance training period, which might be explained by a seasonal impact. However, resistance training did not hinder the decrease in 25-OH D and we find no reason to suggest a positive effect of physical activity on vitamin D status in this patient group. One interesting finding, however, was that even though 25-OH D decreased during the training period, the patients were able to increase their muscle power significantly. Furthermore, we found an unexpected significant correlation between a decrease in 25-OH D and an increase in type 2 muscle fibre size during the training period. Thus, a decrease in 25-OH D during a resistance training period did not limit positive changes in muscle power, physical function, nor muscle hypertrophy in dialysis patients.

These results are limited, however, by the relatively low number of patients. In relation to the data for muscle size, estimations of muscle mass in the lower extremities determined by scanning methods would have been relevant in addition to a measure of muscle size at the fibre level. Scanning methods could have provided additional information of whole leg muscle mass and intramuscular fat content as relevant outcomes in this study.

In conclusion, in a well-nourished sample of dialysis patients, elevated serum IL-6 values were not decreased by a period of resistance training. Half of the patients were found to be suffering from vitamin D deficiency. The low-grade inflammation was associated with reduced muscle power but not with muscle fibre size. Elevated IL-6 values and vitamin D deficiency did not affect the positive effects of resistance training on muscle power and physical function.

## Figures and Tables

**Table 1:** Protocol of 48 exercise sessions covering the 16 weeks of the training.

Exercise	Session 1–6	Session 7–12	Session 13–24	Session 25–36	Session 37–42	Session 43–48
Leg press (sets · reps)	4 · 15	5 · 15	5 · 12	5 · (8–10)	5 · (6–8)	4 · (6–8)
Knee extension (sets · reps)	3 · 15	3 · 15	4 · 12	5 · (8–10)	5 · 8	4 · 8
Knee flexion (sets · reps)	3 · 15	3 · 15	4 · 12	4 · 10	4 · 8	4 · 8

Reps: repetitions.

**Table 2:** Baseline characteristics of the participants.

Parameter	Initial cohort *n* = 36
Age (years)	54 ± 2
Gender (f/m)	15/21
Duration of chronic dialysis (years)	5.4 ± 0.9
Dialysis modality (HD/PD)	30/6
Body mass index (kg/m^2^)	25.0 ± 0.9
Albumin (g/L)	41.2 ± 0.6
Haemoglobin (mmol/L)	7.4 ± 0.2
C-reactive protein (mg/L)	7.2 ± 1.2
Bicarbonate (mmol/L)	25.7 ± 0.6
Comorbidities (score 0–3)	2.3 ± 0.1
Primary renal disease	
T2DM	1
Hypertension	4
Polycystic	8
Glomerulonephritis	14
Nephrosclerosis	2
Other/unknown	6
Interleukin-6 (pg/mL)	8.6 ± 1.3
1,25-OH_2_ D (pmol/L)	31.4 ± 1.8
25-OH D (nmol/L)	60.3 ± 6.0
Normal vitamin D, 25-OH D ≥ 50 nmol/L	49% (*n* = 17)
Mild vitamin D deficiency, 25-OH D 25–50 nmol/L	34% (*n* = 12)
Moderate vitamin D deficiency, 25-OH D 12.5–25 nmol/L	14% (*n* = 5)
Severe vitamin D deficiency, 25-OH D < 12.5 nmol/L	3% (*n* = 1)

F: female; M: male; HD: haemodialysis; PD: peritoneal dialysis. Data are presented as the mean ± SEM, count, or as a percentage.

**Table 3:** Baseline: interleukin-6 split on the median alongside the age, nutritional status, muscle function, and muscle morphology of the participants.

Variable	IL-6 < 6.49 (pg/mL) (*n* = 17)	IL-6 ≥ 6.49 (pg/mL) (*n* = 17)	*P*
*N*	17	17	
Age (years)	49.0 ± 3.4	61.2 ± 2.8	0.024
Gender, male (%)	52.9	64.7	0.486
Albumin (g/L)	42.2 ± 0.9	40.1 ± 0.7	0.068
Energy intake (kcal/kg/day)	2345 ± 132	2220 ± 150	0.710
Protein intake (g/kg/day)	1.49 ± 0.09	1.15 ± 0.11	0.019
Muscle power			
Leg extension, left (W/kg)	2.37 ± 0.20	1.42 ± 0.11	<0.001
Leg extension, right (W/kg)	2.43 ± 0.21	1.59 ± 0.11	0.002
Muscle fibre type			
Type 1 (%)	48.4 ± 3.3	46.5 ± 4.9	0.747
Type 2A (%)	36.3 ± 2.9	37.2 ± 3.1	0.826
Type 2X (%)	15.3 ± 3.5	16.3 ± 3.7	0.935
Muscle fibre size			
Type 1 (*μ*m^2^)	4406 ± 348	4610 ± 320	0.567
Type 2 (*μ*m^2^)	3526 ± 277	3270 ± 257	0.504

Data are presented as the mean ± SEM or as a percentage.

**Table 4:** Baseline: 25-OH D status and age, gender, nutritional status, glucose tolerance, muscle power, and muscle morphology for the participants.

Variable	Normal 25-OH D (≥50 nmol/L)	25-OH D deficiency (<50 nmol/L)	*P*
*N*	17	18	
Age (years)	51 ± 4	55 ± 4	0.525
Gender, male (%)	52	61	0.684
Tested during a summer period (%)	55.6	75.0	0.236
Albumin (g/L)	42.8 ± 0.7	40.1 ± 0.8	0.008
Energy intake (kcal/kg/day)	32.1 ± 2.8	29.9 ± 1.9	0.750
Protein intake (gr/kg/day)	1.39 ± 0.14	1.35 ± 0.10	0.512
Muscle power			
Leg extension, left (W/kg)	2.0 ± 0.3	2.0 ± 0.2	0.216
Leg extension, right (W/kg)	2.0 ± 0.3	2.2 ± 0.2	0.379
Muscle fibre type			
Type 1 (%)	46 ± 4	45 ± 5	0.491
Type 2A (%)	45 ± 3	33 ± 3	0.053
Type 2X (%)	9 ± 2	22 ± 4	0.022
Muscle fibre size			
Type 1 (*μ*m^2^)	4071 ± 256	5000 ± 366	0.048
Type 2 (*μ*m^2^)	3783 ± 276	3397 ± 266	0.950

Data are presented as correlation coefficients, the mean ± SEM, or a percentage.

**Table 5:** Interleukin-6 and 25-hydroxyvitamin D (25-OH D) levels for patients during the study.

Variables	Baseline	Pretraining	Posttraining	*P* control period	*P* training period	*P* in-between periods
IL-6 (pg/mL)	8.8 ± 1.9	14.5 ± 3.3	13.0 ± 3.6	0.063	0.394	0.289
25-OH D (nmol/L)	62.5 ± 8.4	69.1 ± 7.4	59.2 ± 7.5	0.145	0.038	0.167

Data are presented as the mean ± SEM (*n* = 23). The control period was between baseline and pretraining; training period was between pre- and posttraining.

## References

[1] Roubicek T, Bartlova M, Krajickova J (2009). Increased production of proinflammatory cytokines in adipose tissue of patients with end-stage renal disease. *Nutrition*.

[2] Bergström J, Lindholm B, Lacson E (2000). What are the causes and consequences of the chronic inflammatory state in chronic dialysis patients?. *Seminars in Dialysis*.

[3] Zhang W, He J, Zhang F (2013). Prognostic role of C-reactive protein and interleukin-6 in dialysis patients: a systematic review and meta-analysis. *Journal of Nephrology*.

[4] Bologa RM, Levine DM, Parker TS (1998). Interleukin-6 predicts hypoalbuminemia, hypocholesterolemia, and mortality in hemodialysis patients. *American Journal of Kidney Diseases*.

[5] Pedersen BK (2011). Muscles and their myokines. *The Journal of Experimental Biology*.

[6] Drechsler C, Verduijn M, Pilz S (2011). Vitamin D status and clinical outcomes in incident dialysis patients: results from the NECOSAD study. *Nephrology Dialysis Transplantation*.

[7] González EA, Sachdeva A, Oliver DA, Martin KJ (2004). Vitamin D insufficiency and deficiency in chronic kidney disease: a single center observational study. *American Journal of Nephrology*.

[8] Chonchol M, Scragg R (2007). 25-Hydroxyvitamin D, insulin resistance, and kidney function in the Third National Health and Nutrition Examination Survey. *Kidney International*.

[9] Heaf JG, Molsted S, Harrison AP, Eiken P, Prescott L, Eidemak I (2010). Vitamin D, surface electromyography and physical function in uraemic patients. *Nephron—Clinical Practice*.

[10] Gordon PL, Sakkas GK, Doyle JW, Shubert T, Johansen KL (2007). Relationship between vitamin D and muscle size and strength in patients on hemodialysis. *Journal of Renal Nutrition*.

[11] Wassner SJ, Li JB, Sperduto A, Norman ME (1983). Vitamin D deficiency, hypocalcemia, and increased skeletal muscle degradation in rats. *The Journal of Clinical Investigation*.

[12] Molsted S, Harrison AP, Eidemak I, Andersen JL (2013). The effects of high-load strength training with protein- or nonprotein-containing nutritional supplementation in patients undergoing dialysis. *Journal of Renal Nutrition*.

[13] Miskulin DC, Athienites NV, Yan G (2001). Comorbidity assessment using the index of coexistent diseases in a multicenter clinical trial. *Kidney International*.

[14] Toft U, Kristoffersen L, Ladelund S (2008). Relative validity of a food frequency questionnaire used in the Inter99 study. *European Journal of Clinical Nutrition*.

[15] Rikli RE, Jones CJ (2001). *Senior Fitness Test Manual*.

[16] Brooke MH, Kaiser KK (1970). Muscle fiber types: how many and what kind?. *Archives of Neurology*.

[17] Andersen JL, Aagaard P (2000). Myosin heavy chain IIX overshoot in human skeletal muscle. *Muscle & Nerve*.

[18] Højskov CS, Heickendorff L, Møller HJ (2010). High-throughput liquid-liquid extraction and LCMSMS assay for determination of circulating 25(OH) vitamin D3 and D2 in the routine clinical laboratory. *Clinica Chimica Acta*.

[19] The International Society of Nephrology KDIGO, 2012 clinical practice guideline for the evaluation and management of chronic kidney disease.

[20] Molsted S, Harrison AP, Eidemak I, Dela F, Andersen : JL (2013). Improved glucose tolerance after high-load strength training in patients undergoing dialysis. *Nephron Clinical Practice*.

[21] Kaizu Y, Ohkawa S, Odamaki M (2003). Association between inflammatory mediators and muscle mass in long-term hemodialysis patients. *American Journal of Kidney Diseases*.

[22] Cheema BSB, Abas H, Smith BCF (2011). Effect of resistance training during hemodialysis on circulating cytokines: a randomized controlled trial. *European Journal of Applied Physiology*.

[23] Bailey JL, Zheng B, Hu Z, Price SR, Mitch WE (2006). Chronic kidney disease causes defects in signaling through the insulin receptor substrate/phosphatidylinositol 3-kinase/Akt pathway: implications for muscle atrophy. *Journal of the American Society of Nephrology*.

[24] Lim VS, Ikizler TA, Raj DSC, Flanigan MJ (2005). Does hemodialysis increase protein breakdown? Dissociation between whole-body amino acid turnover and regional muscle kinetics. *Journal of the American Society of Nephrology*.

[25] Siew ED, Pupim LB, Majchrzak KM, Shintani A, Flakoll PJ, Ikizler TA (2007). Insulin resistance is associated with skeletal muscle protein breakdown in non-diabetic chronic hemodialysis patients. *Kidney International*.

[26] Kopple JD, Wang H, Fournier M (2006). Transcriptional levels of growth factors in skeletal muscle of maintenance hemodialysis patients. *Journal of Renal Nutrition*.

[27] May RC, Kelly RA, Mitch WE (1986). Metabolic acidosis stimulates protein degradation in rat muscle by a glucocorticoid-dependent mechanism. *The Journal of Clinical Investigation*.

[28] Bruunsgaard H (2005). Physical activity and modulation of systemic low-level inflammation. *Journal of Leukocyte Biology*.

[29] Balducci S, Zanuso S, Nicolucci A (2010). Anti-inflammatory effect of exercise training in subjects with type 2 diabetes and the metabolic syndrome is dependent on exercise modalities and independent of weight loss. *Nutrition, Metabolism and Cardiovascular Diseases*.

[30] Castaneda C, Gordon PL, Parker RC, Uhlin KL, Roubenoff R, Levey AS (2004). Resistance training to reduce the malnutrition-inflammation complex syndrome of chronic kidney disease. *American Journal of Kidney Diseases*.

[31] Yeh S-S, Marandi M, Thode HC (2010). Report of a pilot, double-blind, placebo-controlled study of megestrol acetate in elderly dialysis patients with cachexia. *Journal of Renal Nutrition*.

[32] Wilund KR, Tomayko EJ, Wu P-T (2010). Intradialytic exercise training reduces oxidative stress and epicardial fat: a pilot study. *Nephrology Dialysis Transplantation*.

[33] Raj DSC, Sun Y, Tzamaloukas AH (2008). Hypercatabolism in dialysis patients. *Current Opinion in Nephrology and Hypertension*.

[34] Anand S, Kaysen GA, Chertow GM (2011). Vitamin D deficiency, self-reported physical activity and health-related quality of life: the Comprehensive Dialysis Study. *Nephrology Dialysis Transplantation*.

